# Consensus of the Hellenic Headache Society on the diagnosis and treatment of migraine

**DOI:** 10.1186/s10194-019-1060-6

**Published:** 2019-12-13

**Authors:** Evangelos Kouremenos, Chrysa Arvaniti, Theodoros S. Constantinidis, Ermioni Giannouli, Nikolaos Fakas, Themistoklis Kalamatas, Evangelia Kararizou, Dimitrios Naoumis, Dimos D. Mitsikostas, Hellenic Headache Society

**Affiliations:** 1 Neurology Department, 251 Air Force General Hospital, Athens, Greece; 2 Second Neurology Department, School of Medicine, National & Kapodistrian University of Athens, Attikon Hospital, Athens, Greece; 3 Private Headache Office, Korinthos, Greece; 4 Department of Neurology, Athens Medical Center, Athens, Greece; 5 Neurology Department, 401 Military General Hospital, Athens, Greece; 6 First Neurology Department, School of Medicine, National & Kapodistrian University of Athens, Aeginition Hospital, 72-74 Vl Sofia’s Avenue, 11528 Athens, Greece

**Keywords:** Hellenic headache society, Consensus, Migraine, Treatment

## Abstract

More than 0.6 million people suffer from disabling migraines in Greece causing a dramatic work loss, but only a small proportion of migraineurs attend headache centres, most of them being treated by non-experts. On behalf of the Hellenic Headache Society, we report here a consensus on the diagnosis and treatment of adult migraine that is based on the recent guidelines of the European Headache Federation, on the principles of Good Clinical Practice and on the Greek regulatory affairs. The purposes are three-fold: (1) to increase awareness for migraine in Greece; (2) to support Greek practitioners who are treating migraineurs; and (3) to help Greek migraineurs to get the most appropriate treatment. For mild migraine, symptomatic treatment with high dose simple analgesics is suggested, while for moderate to severe migraines triptans or non-steroidal anti-inflammatory drugs, or both, should be administered following an individually tailored therapeutic strategy. A rescue acute treatment option should always be advised. For episodic migraine prevention, metoprolol (50–200 mg/d), propranolol (40–240 mg/d), flunarizine (5–10 mg/d), valproate (500–1800 mg/d), topiramate (25–100 mg/d) and candesartan (16–32 mg/d) are the drugs of first choice. For chronic migraine prevention topiramate (100-200 mg/d), valproate (500–1800 mg/d), flunarizine (5–10 mg/d) and venlafaxine (150 mg/d) may be used, but the evidence is very limited. Botulinum toxin type A and monoclonal antibodies targeting the CGRP pathway (anti-CGRP mAbs) are recommended for patients suffering from chronic migraine (with or without medication overuse) who failed or did not tolerate two previous treatments. Anti-CGRP mAbs are also suggested for patients suffering from high frequency episodic migraine (≥8 migraine days per month and less than 14) who failed or did not tolerate two previous treatments.

## Introduction

Migraine is the third most prevalent disorder in the world [1] and the first cause of disability in under 50s [2]. Almost three billion individuals were estimated to have migraine or tension-type headache (TTH) in 2016 (1·89 billion with tension-type headache and 1·04 billion with migraine). However, because migraine has a much higher disability burden than TTH, migraine caused 45·1 million (95% UI 29·0–62·8) and TTH only 7·2 million (95% UI 4·6–10·5) YLDs (Years of Live lived with Disability) globally in 2016 [1]. In Europe, the mean per-person annual costs were €1222 for migraine and €3561 for medication-overuse headache, that usually comorbid with chronic migraine, indicating that migraine is a prominent health-related driver of economic losses for the European Union [3]. In Greece about 610.000 suffer from disabling migraines and 283.500 from disabling TTH. Headache sufferers report headache-induced reductions in performance around 3 days/month and only one out of five seek professional treatment for headaches, a private neurologist most often. Interestingly, the vast majority of Greek headache sufferers have never taken prophylaxis for headaches [4]. These findings prompted Hellenic Headache Society (HHS) to initiate a network of headache centres that should manage headache sufferers appropriately. In this light, the consensus outlined here represents an additional effort of the HHS to provide a practical tool including both established and novel treatments for headache and migraine in particular, which can easily be used by Neurologists, General Practitioners and any other health professional involved in headache medicine.

## Methods

The present consensus was drafted by a group of members of the Hellenic Headache Society, in three consecutive meetings followed by four email rounds to establish the final document which was approved by all members of the panel. The recommendations of the European Headache Federation (EHF) for technical investigation for primary headache disorders [5] and for treatment of migraine [6, 7] were the bases for adaption into the national environment. The European Federation of Neurological Societies (EFNS) recommendations for migraine [8] and medication overuse headache [9] were reviewed and additional literature search was performed for updating the treatments, following the methodology described by EFNS task forces. The patients’ preferences obtained in a previous study performed by the HHS [10] were also taken into consideration, together with the National Pharmacy Affairs rules for treatment reimbursement. Migraine diagnosis and classification follows ICHD-3 [11].

## Migraine diagnosis

The diagnosis of migraine is based on the history given by the patient, as well as the normal clinical examination. Only in selected cases is brain imaging required (Table 1) [5]. As no biomarkers exist, taking the patient’s medical history remains the most important part of the diagnostic procedure. The presence of particular features often requires paraclinical evaluation to exclude secondary headaches (Table 2). The HHS advises the use of a semi-structured interview to avoid forgetting important information leading to misdiagnosis. An electronic patient interview and database has been developed by the HHS and is available for headache centers and other physicians who treat headaches in Greece to interview and follow up headache sufferers. Briefly, a number of questions, regarding headache onset, duration, localization, severity (rated in a 11 point visual analogue scale-VAS) and frequency, the pain characteristics, the pattern and the possible trigger causes of headache should be routinely asked. Specific scales to scan for potential affective disorders, e.g. the Hamilton depression and anxiety rating scale are significant [12], together with a list of questions related with disorders of the other body systems. Scales for quality of life changes due to headache (e.g. the Migraine Disability Assessment Test- MIDAS [13], the Headache Impact Test-HIT-6 [14]), that also rate the headache impact and severity are recommended for both the first visit and the follow-up. Likewise, a patient’s headache diary is of great importance for both diagnosis and follow-up. Headache diary, MIDAS (validated in Greeks [13]), HIT-6 [14] (not validated, translated into Greeks) and Hamilton scales (validated in Greeks [15]), are all available upon request from the HHS. Apart from the detailed interview the complete neurological examination should be normal. Physical examination, including blood pressure, body mass index calculation and fundoscopy for papilledema are recommended as well.

**Table 1:** Tests recommended for migraine

ICHD-3 code	Migraine Subtype	Recommended tests
1.1	Migraine without aura	None
	Frequent Episodic Migraine	Brain MRI^a^ Carodit ultrasound or MRA^a^ ESR^a^
1.2	Migraine with aura	Brain MRI^a^
1.2.2	Migraine with brainstem aura	Brain MRI & MRA EEG^a^ Carotid and vertebral arteries ultrasound/or CT or MRA^a^ Genetic evaluation^a^
1.2.3	Hemiplegic migraine	Brain MRI & MRA Genetic evaluation
1.2.4	Retinal migraine	Brain MRI & MRA Ophthalmologic evaluation
1.3	Chronic migraine	Brain MRI Gd & MRV^a^ Fundoscopy^a^ Lumbar puncture Polysomnography^a^
1.4	Complications of migraine	Brain MRI
1.4.2	Persistence aura without infraction	Emergency brain CT or MRI (Carotid and vertebral arteries ultrasound/or CT or MRA ESR^a)^
1.4.3	Migrainous infarction	Emergency brain CT or MRI (Carotid and vertebral arteries ultrasound/or CT or MRA^a)^ ESR^a^
1.4.4	Migraine aura-triggered seizures	Repetitive EEGs or video EEG
1.5	Probable migraine	Brain MRI^a^
1.6	Episodic syndromes that may be associated with migraine	Gastric work-up

Adapted from Mitsikostas et al., 2015 [5] with changes

^a^Indicates specific conditions; *CT* Computerized tomography, *MRI* Magnetic resonance imaging, *MRA* Magnetic resonance angiography, *MRV* Magnetic resonance venography, *Gd* Gadolinium, *EEG* Electro-encephalogram, *ESR* Eryhtrocyte sedimentation rate

**Table 2:** Clinical features warning of a possible underlying disorder

• Headache that peaks in severity in less than five minutes	
• New headache type versus a worsening of a previous headache	
• Change in previously stable headache pattern (e.g. progressive headache, worsening over weeks or longer)	
• Headache that changes with posture (e.g. standing up)	
• Headache awakening the patient	
• Headache precipitated by physical activity or Valsalva manoeuvre (e.g. coughing, laughing, straining)	
• First onset ≥50 years of age	
• Focal neurological symptoms or signs	
• Trauma	
• Fever	
• Seizures	
• History of malignancy	
• History of HIV or active infections	

Adapted from Mitsikostas et al., 2015 [5] with changes

## Treatment of migraine

The management of migraine is multidisciplinary involving pharmaceutical and non-pharmaceutical procedures that are all important and necessary. Here we report suggestions for the pharmaceutical treatment of migraine that is divided into symptomatic and prophylactic. We also cover the potential use of neurostimulation and food supplements as treatment options. No safety issues are covered but we address, of course, particular considerations when it is appropriately significant.

### Symptomatic treatment of migraine

Patients should be advised to receive the symptomatic treatment as soon as they are sure that they are experiencing a migraine attack. Intake of acute medications for migraine must not exceed the 10 days per month for ergotamine, triptans or combinations of drugs, or the 15 days per month for NDAIDs, paracetamol and aspirin, to avoid medication overuse headache. However, we suggest stricter limits to safeguard more (see Medication Overuse Headache Section). Simple analgesics (paracetamol and aspirin, alone, or in combinations, with or without caffeine) are recommended at high doses (e.g. 1 g of paracetamol, or aspirin, per os) for acute treatment of migraines that are typically mild in severity and duration (e.g. rating less than 6/10 in VAS and lasting less than 12 h) [8]. Before intake of non-steroidal anti-inflammatory drugs (NSAIDs) and triptans, oral metoclopramide or domperidone may be useful when migraineurs experience nausea in particular. For the mild to severe migraine attacks, oral NSAIDs and triptans are suggested [8]. There is good documentation (level A) for naproxen (500-1000 mg), ibuprophen (200-800 mg) and diclofenac (50-100 mg), and for tolfenamic acid (200 mg, level B). We do not suggest following the concept of stratified treatment as EFNS Task Force suggests, however [8]. We believe that a therapeutic plan tailored to the individual patient is more efficient, saves time and fits better to the good clinical practice rules. The personal medical history of each patient will guide the physician to the right decision-making. For severe migraine attacks (e.g. menstrual migraine) subcutaneous sumatriptan is recommended.

Headache may recur within 24 or even within 48 h after successful treatment (meaning pain free 2 h post treatment) with triptans [16]. One to four out of 10 patients taking an oral triptan experience recurrence [16]. A second dose of the triptan is often effective but only in case the headache recurs. On the contrary, physicians should clarify to their patients that if the first dose of a triptan does not work, a second dose is useless [17]. Interestingly, there is good documentation (level A) that combining a NSAID with a triptan (naproxen 500 mg with sumatriptan 85 mg, or sumatriptan 100 mg since in Greece this dosage is not available) reduces headache recurrence and improves efficacy [18–20]. Not all patients are candidates for all of the above treatment suggestions, since there are several contraindications and significant safety issues [21] that this report does not cover (Table 3).

**Table 3:** Use of simple analgesics, NSAIDs, triptans or combinations for the symptomatic treatment of migraine

• For decision-making physicians should take into account the patients’ preferences, life-style particularities and personal expectancies.	
• In patients with mild migraines we suggest using aspirin or paracetamol 1 g, per os*,* for the symptomatic treatment of migraine.	
• In patients with migraine who have failed to aspirin or paracetamol 1 g, or who cannot use these agents because of comorbidities, adverse events or poor tolerability, or their migraines have mild to severe intensity, we suggest using triptans per os*,* or non-steroidal anti-inflammatory drugs (NSAIDs), or combinations, according to their potential comorbidity and tolerance to these drugs.	
• If migraines recur after successful symptomatic treatment, the use of long acting triptans may be useful (e.g. almotriptan, naratriptan and fravotriptan).	
• If migraines do not respond to oral high efficacy triptans (e.g. eletriptan 40 or 80 mg, or rizatriptan 10 mg) a combination of a triptan with NSAID, sumatriptan 50 mg or 100 mg with naproxen 500 mg in particular, is recommended.	
• Not all triptans share the same efficacy, safety and pharmacokinetics within an individual, thus no response to one triptan does not predict unresponsiveness to all triptans.	
• Consistency of triptans and other symptomatic drugs for migraine is limited and may vary by individual; therefore, rescue medication should be offered to all patients for symptomatic treatment of migraine; this should be parenterally given to avoid potential gastric stasis, e.g. sumatriptan subcutaneously, or NSAID suppositories.	
• All migraineurs should be educated by their treating physicians to limit the use of symptomatic anti-migraine drugs to a maximum of two days per week if they use triptans, or combinations of drugs, or three days per week if they use NSAIDs or simple analgesics, in order to avoid medication overuse headache (since the related ICHD-3 limits are 10 and 15 days/month [11], by this suggestion we prevent from medication overuse securely).	
• All migraineurs should be educated by their treating physicians that the symptomatic treatment of migraine is only a small part of the management of migraine, that requires additional prophylactic pharmacotherapy in most cases, life-style changes and non-pharmaceutical approaches depending on the particular patient and the potential comorbidity.	

Because the consistency of all available symptomatic treatments is limited, all migraineurs should be advised for rescue treatment. The HHS emphatically recommends this in order to limit both the unnecessary hospital admissions and the patients’ long suffering. Rescue treatment should be offered when 2 h post treatment there is no pain relief and should be parenteral for quicker drug absorption and efficacy and because at this late stage of a migraine attack gastric stasis may occur. Apart from subcutaneous sumatriptan, the NSAIDs suppositories are suggested, since no other formulations are currently available in Greece. Intramuscular diclofenac 75 mg may be given as rescue medication at home [22], as well. Status migrainosus can be treated by intravenous cortoicosteroids, although this has not universally been proved to be helpful [8]. Other options include intravenous valproate [23] and a combination of intravenous tramadol with paracetamol [24]. Intravenous metoclopramide alone does not seem to help [25], although there is good evidence for a combination of metoclopramide 10 mg and aspirin 1 g for the symptomatic treatment of migraine, given per os, at home [26]. Because of lack of evidence demonstrating efficacy and major concern about sub-acute or long-term sequelae, injectable morphine and hydromorphone should best be avoided [27]. Generally, HHS suggests avoiding the use of narcotics for migraine treatment, either for acute or for prophylactic treatment (with the exception of tramadol in a hospital setting, in a few selected cases).

### Prophylactic treatment of migraine

When migraine is disabling and/or the attacks are intractable, prophylactic treatment is suggested. The prevention of migraine chronification is the most important part of migraine management and includes not only the pharmaceutical treatment of migraine but also the treatment of potential comorbidities, such as depression-like conditions and obesity [28]. There is not a cut off threshold of the number of migraine days per month that requires prophylactic treatment. The treating physician decides along with the patient upon the disability and the comorbidities that the specific patient may suffer from. To make a more tailored choice of medication for migraine prevention, HHS suggests the sub-classification of episodic migraine into low (< 8 migraine days per month) and high frequency (8–14 migraine days per month) episodic migraine. By this sub-classification the new emerging treatments (e.g. the anti-CGRP monoclonal antibodies) fit better to the suggested therapeutic algorithm.

### Episodic migraine

For the prevention of episodic migraine (EM) the HHS accepts the EFNS task force recommendations [8]. The drugs of first choice with level A documentation are metoprolol (50–200 mg/d), propranolol (40–240 mg/d), flunarizine (5–10 mg/d), valproate (500–1800 mg/d) and topiramate (25–100 mg/d). Drugs of second choice with level B documentation are amitriptyline (50–150 mg/d), venlafaxine (75–150 mg/d), naproxen (500–1000 mg/d) and new purified formulation of petasites (150 mg/d). Angiotensin II receptor blocker candesartan (16-32 mg/d) should be added to the list of first choice medications [29, 30]. There is margnial documentation for several other agents that have been tested for the prevention of EM (third choice drugs) [8]. For the high frequency EM prevention, HHS follows the EHF recommendations and suggests the use of anti-CGRP monoclonal antibodies (anti-CGRP mAbs) as second choice after the failure of two first choice agents (Table 4).

**Table 4:** Use of anti-CGRP monoclonal antibodies for migraine prevention

• In patients with high frequency episodic migraine who have failed at least two of the available medical treatments or who cannot use other preventive treatments because of comorbidities, adverse events or poor compliance, we suggest the use of erenumab, fremanezumab, or galcanezumab;	
• In patients with chronic migraine who have failed at least two of the available medical treatments or who cannot use other preventive treatments because of comorbidities, adverse events or poor compliance, we suggest the use of erenumab, fremanezumab, or galcanezumab;	
• In patients with high frequency episodic migraine, before starting erenumab, galcanezumab or fremanezumab we suggest to stop oral preventive drugs unless the patient had a previous history of chronic migraine before prevention; in this case, we suggest adding the anti-CGRP monoclonal antibody to the ongoing treatment and to re-assess the need of treatment withdrawal;	
• In patients with chronic migraine who are on treatment with any oral drug with inadequate treatment response we suggest adding erenumab, fremanezumab, or galcanezumab and considering later withdrawal of the oral drug. In patients with chronic migraine who are on treatment with onabotulinumtoxinA with inadequate treatment response we suggest stopping onabotulinumtoxinA before initiation of erenumab, fremanezumab, or galcanezumab. In patients with chronic migraine who are on treatment with erenumab, fremanezumab, or galcanezumab and who may benefit from additional prevention we suggest adding oral preventive drugs;	
• In patients with high frequency episodic or chronic migraine, we suggest considering pausing treatment with erenumab, fremanezumab, and galcanezumab after 6–12 months of treatment in order to evaluate the migraine status; if migraines recur to the pre-treatment status or to an undesirable level for the patient, treatment re-administration is suggested.	
• In patients with chronic migraine and medication overuse, we suggest using erenumab, fremanezumab, and galcanezumab before or after withdrawal of acute medications;	
• In patients with migraine, we suggest avoiding anti-CGRP monoclonal antibodies in pregnant or nursing women, in individuals with alcohol or drug abuse, cardio and cerebrovascular diseases, and with severe mental disorders;	
• In patients with migraine on treatment with anti-CGRP monoclonal antibodies, there is no need for testing for binding and/or neutralizing antibodies.	

Adapted from Sacco et al., 2019 [6] with changes

### Chronic migraine

For the prevention of chronic migraine (CM), HHS suggests the use of first choice drugs recommended for the prevention of EM, with emphasis to topiramate, valproate, flunarizine and venlafaxine. Topiramate 100 mg/d has little documentation for CM [31, 32]. In addition it can be used as a stabiliser for mood disorders [33] like valproate [34], but the latter has, or should have, very limited use in young females [35]. Venlafaxine is a typical antidepressant and affective disorders are very often comorbid with chronic migraine and/or MOH [12], thus its use could be helpful when anxiety and/or depressive symptoms are present in patients with CM. Flunarizine showed efficacy in CM in one small size, open label, trial [36]. No combination of topiramate and propranolol is recommended for the prophylactic treatment of CM [37]. However, botulinum toxin type A (BTXA) and the anti-CGRP mAbs share the best documentation for the prevention of CM (level A). Following the EHF recommendations [6, 7], the HHS suggests the use either of BTXA, or anti-CGRP mAbs (erenumab, fremanezumab, or galcanezumab), for those patients who have tried two previous prophylactic treatments for CM (topiramate and flunarizine and/or venlafaxine when depression like symptoms are detected), but they did not tolerate them, or their migraines did not improve. For the use of anti-CGRP mAbs and BTXA in CM see Tables 4 and 5, respectively. Both drug classes are safe and well tolerated [38–40]. Notably, the documentation of the first line suggested prophylactic treatments for CM is very poor or even empirical. However, the Greek Authorities demand failure to first line prophylactic treatments to reimburse BTXA. In order for the Greek doctors to comply with this requirement, they may use topiramate (100-200 mg/d), flunarizine (10 mg/d) or venlafaxine (150 mg/d) as first line treatments.

**Table 5:** Use of botulinum toxin A (BTXA) in chronic migraine

• BTXA should be administered according to the PREEMPT injection protocol, i.e. injecting 155 U–195 U to 31–39 sites every 12-weeks	
• Treatment with BTXA should be stopped, if the patient does not respond to the first three treatment cycles.	
• Patients treated with BTXA are defined as non-responders, if they have less than 30% reduction in headache days per month during treatment.	
• Evaluate the response to continued treatment with BTXA on the basis of headache calendars by comparing the 4 weeks before and 4 weeks after each treatment cycle.	
• Stop treatment in patients with a reduction to less than 10 headache days per month for 3 months (other factors such as headache intensity, disability and patient preferences should also be considered).	
• Re-evaluate 4–5 months after stopping BTXA to secure that the patient has not returned to CM.	
• In patients with chronic migraine and medication overuse, we suggest using BTXA, before or after withdrawal of acute medications, as second-line prophylactic treatment.	

Adapted from Bendtsen et al., 2018 [7] with changes

### Medication overuse headache

Medication overuse headache (MOH) occurs by definition in chronic migraine [11], although drug overuse may occur in patients suffering from high frequency episodic migraine as well. Prevention of migraine chronification stands as the most important issue in migraine management, as stated previously. As soon as MOH appears immediate treatment is required, which includes patient education to reduce medication intake, discontinuation of the overused medication and individual prophylactic treatment [9, 41]. Whether abrupt withdrawal or tapering down of overused medication is better depends on the patient’s personality and potential comorbidities he/she may suffer from and it is not related to outcome [9]. Inpatient withdrawal therapy may be needed, in particular for patients overusing opioids, benzodiazepines, or barbiturates [9] (Table 6).

**Table 6:** Management of Medication Overuse Headache (MOH)

• For decision-making physicians should take into account the patients’ preferences, life-style particularities and personal expectancies.	
• Physicians should educate patients for the mechanisms underlying the pathophysiology of MOH and the ways the patients should avoid overusing medications for treating their migraines.	
• Depending on the patients’ personality, individual preferences and comorbidity, acute withdrawal or tapering down of the drug overuse is recommended.	
• Immediate prophylactic treatment together with the drug overuse withdrawal or tapering down, is recommended as well, including topiramate 100-200 mg/day and prednisone or prednisolone 60 mg/d. The use of botulinumtoxinA and/or anti-CGRP monoclonal antibodies (erenumab, fremanezumab, and galcanezumab) is recommended, as second-line treatment.	
• Not only migraine, but all potential comorbidities should also be treated.	
• To treat anxiety and/or depression like symptoms that are highly likely to co-occur with MOH, venlafaxine 150 mg/df is recommended.	
• Inpatient withdrawal therapy may be needed, in particular for patients overusing opioids, benzodiazepines, or barbiturates.	
• Because the prognosis of MOH is poor and the relapse rate high, close follow-up of patients is recommended.	

There is moderate evidence for the prophylactic treatment in patients with CM and medication overuse with topiramate up to 200 mg [9]. Corticosteroids (at least 60 mg prednisone or prednisolone) and amitriptyline (up to 50 mg) are possibly effective in the treatment of withdrawal symptoms [9]. However, prophylactic treatment with BTXA and anti-CGRP mAbs also improve MOH and should be suggested [6, 7] (Tables 4 and 5) in patients who do not need hospitalization. Not only migraine, but all potential comorbidities should be evaluated and treated accordingly to prevent from MOH recurrence. Because anxiety and depression like symptoms are very common in patients overusing drugs for symptomatic treatment of migraine [12], treatment with venlafaxine 150 mg/d that also improves both TTH [42, 43] and migraine may be helpful. Non-pharmaceutical interventions, including cognitive behavioral treatment are also recommended. A large proportion of patients suffering from MOH often relapse [41] thus close follow-up is recommended.

### Neurostimulation for migraine treatment

Neurostimulation is a novel promising therapeutic approach that helps patients with migraine. Patients in Greece prefer this approach in particular [10] and treating physicians should be aware of these techniques that are divided into invasive and non-invasive approaches. Here we discuss the application only of the non-invasive devices, since the invasive ones require management in a tertiary headache center exclusively [44], such as the vagus nerve stimulation (VNS), the external trigeminal nerve stimulation (eTNS), and the transcranial magnetic stimulation (TMS). In Europe several devices carry the CE mark, among them only four devices have low to moderate evidence for treatment of migraines [45]. The device of the VNS for the symptomatic treatment of migraine [46], the single transcranial magnetic stimulation (sTMS) for the symptomatic treatment of migraine with aura, the repetitive transcranial magnetic stimualtion (rTMS) for the prophylactic treatment of episodic migraine [47] and the eTNS for both the symptomatic and prophylactic treatment of episodic migraine [48, 49]. Thus, migraineurs may use these devices although they are not reimbursed in Greece currently.

### Dietary supplements for migraine treatment

Several randomized clinical trials (RCTs) suggest dietary and vitamin supplementation and different herbs effectiveness in the treatment of migraine, although several methodological shortcomings, especially lack of comparison with standard treatment and placebo, have been noticed [50]. Coenzyme Q10, magnesium citrate, petasites and riboflavin share low to moderate documentation and could be used for migraine prevention when patient preferences exclude pharmacotherapy. There is limited evidence that prophylaxis with combination of simvastatin (40 mg/d) with vitamin D (1000 Units/d) may help patients suffering from episodic migraine [51]. Other combinations may be useful as well, but the evidence for efficacy is limited. When a patient refuses pharmacotherapy, in particular those patients with negative previous experiences or expectations, e.g. patients with nocebo behaviours [52, 53], HHS suggests the use of dietary supplements to improve migraines. Cognitive behavioural treatment is also effective, not only in these patients, but in those co-suffering from any affective disorder as well [54].

## Synopsis

Migraine is a disabling disease requiring effective treatment. Diagnosis of migraine remains clinical and only in a few cases testing is needed to exclude secondary headaches. HHS suggests taking the interview carefully, based in a semi-structured questionnaire, to cover all aspects of headache features and to follow-up patients with headache diaries. Specific scales to assess headache burden and comorbidities are also suggested, along with a specifically designed database for headache follow-up. For therapeutic decision making physicians should take into account the individual migraine impact and/or disability, potential comorbidities and patient’s personal preferences. HHS expresses two major suggestions: adding a rescue medication in the symptomatic migraine treatment and introducing early prophylactic treatment to prevent migraine chronification and medication overuse. For mild migraines, symptomatic treatment with high dose simple analgesics is suggested, while for mild to severe migraines triptans or NSAIDs, or both, should be administered following an individual tailored therapeutic strategy. Rescue medications should be administered parentally (e.g. sumatriptan subcutaneously). For the prevention of episodic migraine, metoprolol (50–200 mg/d), propranolol (40–240 mg/d), flunarizine (5–10 mg/d), valproate (500–1800 mg/d), topiramate (25–100 mg/d) and candesartan (16–32 mg/d) are the drugs of first choice. For chronic migraine prevention topiramate (100-200 mg/d), valproate (500–1800 mg/d), flunarizine (5–10 mg/d) and venlafaxine (150 mg/d) may be used, although the evidence is very limited. Botulinum toxin type A and anti-CGRP mAbs are recommended for patients suffering from chronic migraine (with or without medication overuse) who failed or did not tolerate two previous first choice treatments. Anti-CGRP mAbs are also suggested for patients suffering from high frequency episodic migraine who failed or did not tolerate two previous first choice treatments. Because many migraine sufferers prefer treatment with neurostimulation in Greece, treating physicians should be familiar with these techniques. There is limited but promising evidence for the use of VNS for the symptomatic treatment of migraine, sTMS for the symptomatic treatment of migraine with aura, rTMS for the prophylactic treatment of episodic migraine and eTNS for both the symptomatic and prophylactic treatment of episodic migraine. Dietary supplements may be useful for those refusing pharmacotherapy. Almost all patients would benefit from cognitive behavioural treatment, but only those who prefer and withstand it are good candidates. Safety issues and patients’ adherence is of great importance in treatment monitoring. Overall, migraine is a treatable disease that needs particular care. Patients may benefit only if treated appropriately after medical consultation. For refractory cases physicians may refer their patients to the Headache Centres established by the HHS.

## Data Availability

There are no original data.

## Abbreviations

Anti-CGRP mAbs: Monoclonal antibodies targeting the CGRP pathway
BTXA: Botulinum toxin type A
CGRP: Calcitonin gene-related peptide
CM: Chronic Migraine
EFNS: European Federation of Neurological Societies
EHF: European Headache Federation
EP: Episodic Migraine
eTNS: External trigeminal nerve stimulation
HHS: Hellenic Headache Society
HIT-6: Headache Impact Test
ICHD-3: International Classification of Headache Disorders-3
mAbs: Monoclonal antibodies
MIDAS: Migraine Disability Assessment Test
MOH: Medication overuse headache
NSAIDs: Non-steroidal anti-inflammatory drugs
RCTs: Randomized clinical trials
TMS: Transcranial magnetic stimulation
TTH: Tension-type headache
VAS: Visual analogue scale fro pain
VNS: Vagus nerve stimulation
